# The Step of Incorporation of *Bacillus coagulans* GBI-30 6086 Into “requeijão cremoso” Processed Cheese Does Not Affect Metabolic Homeostasis of Rats

**DOI:** 10.3389/fmicb.2019.02332

**Published:** 2019-10-22

**Authors:** Mariana B. Soares, Valfredo A. Santos-Junior, E. R. Tavares Filho, Pablo C. B. Lollo, Priscila N. Morato, Jaime Amaya-Farfan, Eliene P. R. Pereira, Celso F. Balthazar, Adriano G. Cruz, Rafael C. R. Martinez, Anderson S. Sant’Ana

**Affiliations:** ^1^Department of Food Science, Faculty of Food Engineering, University of Campinas, Campinas, Brazil; ^2^Department of Food and Nutrition, Faculty of Food Engineering, University of Campinas, Campinas, Brazil; ^3^Department of Food Technology, Faculty of Veterinary, Fluminense Federal University, Niterói, Brazil; ^4^Department of Food, Federal Institute of Education, Science and Technology of Rio de Janeiro, Rio de Janeiro, Brazil

**Keywords:** dairy foods, functional foods, diet, dairy consumption, anti-stress system

## Abstract

Dairy product consumption is a common habit in Brazil. These products present a good matrix for probiotic incorporation. Thus, in this study the feasibility of producing a probiotic “requeijão cremoso” incorporated with *Bacillus coagulans* GBI-30 6086 in three different steps and its metabolic effect in an animal model for 2 weeks has been evaluated. Wistar adult health rats were randomized into one to five groups (*n* = 8 for each group): Control (C); “requeijão cremoso” without probiotic (RC); probiotic inoculated in the milk before pasteurization at 65°C/30 min (RPP); “requeijão cremoso” inoculated before the fusion step and consequently exposed to 90°C/5 min (RPF); and “requeijão cremoso” inoculated after fusion step, i.e., once the product temperature reached 50°C (RPAF). At the end of treatment, analysis of molecular markers of proteins of stress and antioxidant system, HSP 25, 60, 70 and 90, SOD and catalase were performed in the animals’ muscles by Western Blot technique. The HSP25, HSP90 and catalase levels of C, RPP, RPF, and RPAF were similar, indicating that the homeostasis remained unchanged. The incorporation of *B. coagulans* GBI-30 6086 in the “requeijão cremoso” was shown to be stable and the microorganism remained viable in all steps tested. The incorporation of the probiotic strain in the fusion stage facilitated the technological process, since it allowed a better homogenization of the product and did not affect the maintenance of the metabolic homeostasis of rats.

## Introduction

Probiotics are well-defined “as living microorganisms which, when administered in adequate amounts, can confer a health benefit to the host” (Hill et al., 2014). Food components are known to interact with probiotic bacteria strains and assist in their colonization, bringing leading beneficial health effects (Desfossés-Foucault et al., 2012; Ranadheera et al., 2012; Jessie-Lau and Chye, 2018; Nambiar et al., 2018; Pérez Montoro et al., 2018; Rabah et al., 2018; da Costa et al., 2019; Majeed et al., 2019). Since food composition is essential for probiotic survival and functionality, it is important to assess the potential health effects of these microorganisms in specific food matrices (Ranadheera et al., 2010). Dairy product consumption is a common habit in Brazil and these foods are considered a good matrix to probiotics (Lollo et al., 2015; Martins et al., 2015, 2018; Dittmann et al., 2017). Therefore, the expansion in the number and diversity of dairy probiotic foods represents an interesting approach to enhance the consumer’s exposure to a variety of probiotics in the diet, consequently aiming to improve host health.

“Requeijão cremoso” comprises a kind of processed cheese broadly manufactured and consumed in Brazil under different formulas and a variety of technologies. The fusion step is key during the production of “requeijão cremoso” as it is aimed to obtain a consistent cheese blend (Cunha et al., 2010; da Cunha et al., 2012). Thus, the use of high temperatures (between 85 and 95°C for up to 5 min) to attain a homogenous cheese emulsion may negatively impact the production of a probiotic “requeijão cremoso” when traditional probiotic strains of *Lactobacillus* and *Bifidobacterium* are employed (Oliveira et al., 2016). While probiotic strains of *Bifidobacterium* and *Lactobacillus* comprise the most studied and used probiotic microorganisms in processed food products worldwide, especially in dairy foods (Esmerino et al., 2015; Dantas et al., 2016; Felicio et al., 2016; Pereira et al., 2016), these microorganisms are susceptible to high temperatures and would possibly not survive under such processing conditions (Tripathi and Giri, 2014; Oliveira et al., 2018a). An alternative, to overcome this issue and allow the incorporation of probiotics in products such as the “requeijão cremoso,” is the use of probiotic strains of *Bacillus* (as spores), that can survive severe processing conditions as well as tolerate and resist against the harsh conditions found in the gastrointestinal tract (Cutting, 2011; Soares et al., 2019a, b). The use of probiotic strains of *Bacillus* can be advantageous due to the long stability of their spores, which will ensure high viability in food products throughout shelf-life (Tripathi and Giri, 2014). An additional advantage of the use of probiotic strains of *Bacillus* is the possibility of using lower effective doses since the spores present higher survival ability (Blanch et al., 2014).

In fact, probiotic *Bacillus* strains have been commonly employed in human medicine for the prevention of digestive problems and treatment of urinary tract infections (Nithya and Halami, 2013). Although the use of probiotic strains of *Bacillus* in food products is still recent (Lee et al., 2017; Jeon et al., 2018; Marcial-Coba et al., 2019; Soares et al., 2019a, b), research has described the health benefits associated with their ingestion (Nyangale et al., 2015; Jäger et al., 2016, 2018; Keller et al., 2017). For instance, probiotic strains of *Bacillus* were shown to stimulate the immune system (Huang et al., 2013; Sassone-Corsi et al., 2016). Other beneficial effects of probiotic *Bacillus* strains comprise the enhancements in carbohydrates and protein absorption (Jäger et al., 2018; Keller et al., 2018), microbiota modulation in elderly (Nyangale et al., 2014, Nyangale et al., 2015), enhancement in the recovery from exercises and decrease in injuries of muscle tissues (Jäger et al., 2016), abdominal discomfort and swelling decrease (Hun, 2009, Kalman et al., 2009), inhibitory properties against pathogens (Fitzpatrick et al., 2011; Honda et al., 2011), anti-obesity effects (Choi et al., 2016; Wang et al., 2019), improvement in digestive health (Rhayat et al., 2019) and anti-diarrhea effects (Urdaci et al., 2018).

The regulation of host immunological response is one of the primary ways probiotic exert effects on human beings (Cunningham-Rundles et al., 2000; Galdeano and Perdigón, 2006; Yan and Polk, 2011). The main functions of the intestinal immune system include the immune-inflammatory response suitable to suppress the action of pathogenic microorganisms or promote resistance to various compounds (de Almada et al., 2015). In reality, probiotics can activate the immune response by contact with several cells present in the intestinal mucosa, including monocytes, macrophages, B, T, NK and dendritic cells (Coppola and Gil-Turnes, 2004; Cano et al., 2013; Lollo et al., 2013b). Moreover, probiotics can induce the expression of cellular heat shock proteins (HSP) that provide higher resistance and tolerance toward several aggressive factors. This system of defense-antioxidant proteins play an important role in protecting and repairing damaged cellular proteins (Petrof et al., 2004). Actually, these substances are activated during episodes of increase in body temperature, lack of control of reactive oxygen species (ROS), ischemia, hypoxia and glucose deficiency, among others (Morimoto, 1998; Belter et al., 2004; Jang et al., 2008; Staib et al., 2009; Lollo et al., 2013b). Under these conditions, structure, integrity and functionality of cells are kept by HSPs (Silver et al., 2012). Thus, probiotics can help maintain the homeostasis of the body against different types of stress conditions.

Therefore, the aim of this study was to assess the feasibility of production of a probiotic “requeijão cremoso” added of *Bacillus coagulans* GBI-30 6086 and to determine the metabolic effect of its acute consumption using an experimental animal model. In this sense, the effect of the consumption of the probiotic “requeijão cremoso” was evaluated using rats through the determination of standard biochemical parameters and molecular markers of stress.

## Materials and Methods

### Probiotic Strain

*Bacillus coagulans* GBI-30 6086 (Ganeden Biotech, Mayfield Heights, United States) is probiotic strain with GRAS (generally recognized as safe) status commercially available in spores’ form. *B. coagulans* GBI-30 6086 transiently colonizes the intestine without the need for frequent consumption (Maathuis et al., 2010; Orrù et al., 2014; Salvetti et al., 2016). Freeze-dried spores of *B. coagulans* GBI-30 6086 were added to samples in order to achieve 10^8^–10^9^ spores per portion of the food product (30 g), i.e., approximately 10^7^–10^8^ spores/g.

### Preparation of “requeijão cremoso”

The “requeijão cremoso” used in the experiments presented the following composition: 40% (w/w) of fresh cheese mass, 37% (w/w) of milk cream with 35% of fat (Atilate, Itatiba, Brazil), 20% (v/w) distilled water, 1.5% (w/w) of emulsifying salt Joha S2 (ICL Food Specialties, São Bernardo do Campo, Brazil) and 1.5% (w/w) of NaCl (Dinâmica, São Paulo, Brazil). The food product was prepared as previously described by Oliveira et al. (2018b). Samples of the “requeijão cremoso” were kept at 6°C throughout the *in vivo* test period.

### *B. coagulans* GBI-30 6086 Strain Count

The enumeration of *B. coagulans* GBI-30 6086 spores in “requeijão cremoso” samples was carried out at the beginning and the end of the study. For this, samples of “requeijão cremoso” were heat shocked at 80°C/10 min. Subsequently, samples were diluted (1:10) in 1% peptone water, following serial decimal dilutions. Then, aliquots of 1 mL were pour plated into Petri dishes containing formulated Agar Glucose Yeast Extract – GYE [BC – Yeast Extract (Oxoid, Basingstoke, United Kingdom) (5 g/L); D-glucose (5 g/L); Peptone (Acumedia, Lansing, United States) (5 g/L); Potassium Phosphate Monobasic (0.5 g/L); Dibasic Potassium Phosphate (0.5 g/L); Magnesium Sulfate (0.3 g/L); Bacteriological Agar (Inlab, São Luis, Brazil) (15 g/L); Sodium Chloride (10 mg/mL); Zinc Sulfate (1.6 mg/mL); and Mineral Solution (1 ml/L – Manganese Sulfate (16 mg/mL); Cobalt Sulfate 7⋅H_2_O (1.6 mg/ml); Copper Sulfate 5⋅H_2_O (1.6 mg/mL); Iron Sulfate 7⋅H_2_O (18 mg/mL)]. All components added to GYE agar were from Dinâmica, São Paulo, Brazil, unless otherwise stated. Plates were incubated at 40°C/48 h as recommended by the supplier of *B. coagulans* GBI-30 6086.

### Animals

For the *in vivo* experimental model, forty male Rattus novergicus, Wistar lineage (free of pathogens, 21 days old) acquired from the Multidisciplinary Center for Biological Research of the University of Campinas (SP, Brazil) were used. Rats were maintained (±22°C, 45–60% RH, 12 h of inverted light cycle) in separate growth enclosures. Animals were provided with feedstuff (Labina, Purina, Brazil) and water *ad libitum* up until they reach adulthood (about 8 weeks of life and ∼300 g of weight). This weight guaranteed an adequate volume of gavage without causing unnecessary stress to animals. The performance of the experiments was approved by the Ethical Committee in the Use of Animals of the University of Campinas (CEUA/UNICAMP) – Protocol # 3444-1.

**FIGURE 1:**
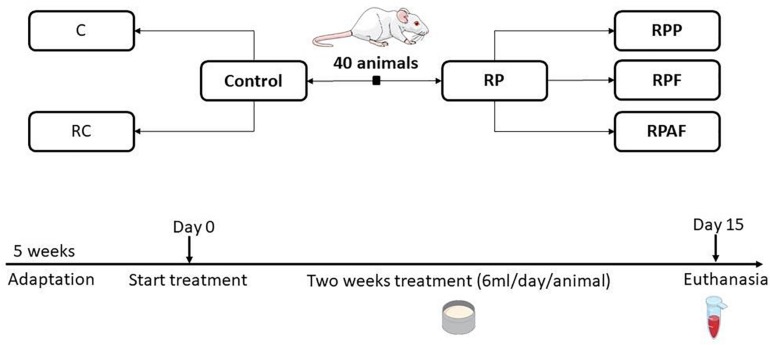
Animals groups and experimental design used in the study. Animals were randomly assigned according to the following groups: Control (C, *n* = 8); Control “requeijão cremoso” cheese (RC, *n* = 8); Probiotic “requeijão cremoso” containing spores of *B. coagulans* GBI-30 6086 inoculated in the milk pasteurization step (RPP, *n* = 8); Probiotic “requeijão cremoso” containing spores of *B. coagulans* GBI-30 6086 inoculated during fusion stage (RPF, *n* = 8); Probiotic “requeijão cremoso” containing spores of *B. coagulans* GBI-30 6086 inoculated after fusion stage (RPAF, *n* = 8). Animals were maintained under adaptation until reaching the adult stage (5 weeks) and then treated with control or probiotic product for 2 weeks. Euthanasia took place on the 15th day after 8 h of fasting.

The experimental design was used to evaluate the effects of a 2-week consumption of the “requeijão cremoso” with *B. coagulans* GBI-30 6086 in health adult rats (Figure 1). Animals were randomly allocated into five groups (*n* = 8 per group): Control (C); “requeijão cremoso” without probiotic (RC); *B. coagulans* GBI-30 6086 inoculated in the milk before pasteurization at 65°C/30 min (RPP); “requeijão cremoso” inoculated with *B. coagulans* GBI-30 6086 before the fusion step and consequently exposed to 90°C/5 min (RPF); and “requeijão cremoso” inoculated with *B. coagulans* GBI-30 6086 after fusion step, i.e., once the product temperature reached 50°C (RAPF).

Commercial feed (Labina, Purina, Brazil) was the basis for feeding for all groups throughout the trial (Table 1). Water and chow were offered *ad libitum*. Treatment intervention and control were administered to animals through an orogastric tube during 2 weeks, as follows: animals were fed daily with 6 mL of the corresponding treatment, divided in two doses of 3 mL per day. After treatment, animals were sacrificed and blood, tissues and organs samples were taken for the performance of biochemical, hematological and molecular analyses.

**TABLE 1:** Mean values (±SD) of commercial diet compounds evaluated for centesimal composition.

	**Commercial diet (g/100 g)**

Humidity	8.7 ± 0.8
Lipids	6.2 ± 0.2
Protein	21 ± 1.1
Ash content	8.3 ± 0.7
Carbohydrates	55.8 ± 0.9
Total	100
	

### Samples Analysis

#### Centesimal Composition

For the evaluation of the centesimal composition of commercial and experimental diets, values of ash content, moisture, and crude protein (Kjeldahl) were determined according to AOAC methodology (AOAC, 2007) and the number of lipids was obtained by the Bligh and Dyer method (Bligh and Dyer, 1959); carbohydrates, in turn, were indirectly estimated by difference.

#### Monitoring of Weight Gain and Commercial Feed Intake

Rats were weighed twice a week, and food consumption control was performed by means of the difference between the amount of feed offered and not consumed by the animals.

#### Biochemical Parameters

After animals’ sacrifice, blood samples were taken using BD-Vacutainers (Becton Dickinson, Franklin Lakes, United States). The biological material was maintained at 4°C and centrifuged (Sigma, Germany) at 3,000 g (4°C, 12 min) to attain serum and assess the rates of glucose, total cholesterol, uric acid, high density lipoprotein (HDL), triacylglycerols, total protein, albumin, alanine aminotransferase (ALT), aspartate aminotransferase (AST), and creatinine kinase (CK). The biochemical parameters evaluated were determined in duplicate. All spectrophotometric analysis were carried out using Laborclin tools (São Paulo, Brazil) and a Biotech Época microplate reader (BioTek, Winooski, United States).

#### Hematologic Parameters

Blood samples were collected with Vacutainer tubes (4 mL) added of EDTA and K3, and subjected to hematological analysis using an automatic cell counter (Ac. T5diff Hematology Analyzer, Beckman Coulter, High Wycombe, United Kingdom). The intra-test factor of variation for entirely determined parameters was less than 3%.

#### Protein Extraction and Immunoblotting

For the molecular analysis of anti-stress and antioxidant proteins system, right and left sore muscles were collected. Analysis of the molecular markers were performed using the Western blot technique, and protein quantification was done according to the Lowry method (Lowry et al., 1951). Briefly, 100 mg of frozen soleus muscle were homogenized in Triton buffer (100 mM Tris, pH 7.4, 1% Triton X-100) added of NaF (100 mM), sodium pyrophosphate (100 mM), Na_3_VO_4_ (10 mM), EDTA (10 mM), aprotinin (0.1 mg/mL) and PMSF (2 mM) as detailed in Lollo et al. (2013b). For the immunoblotting step, SDS-PAGE method was performed using soleus muscle tissues homogenates. Subsequently, the Western Blot Biocom Western system was used to transfer proteins bands to a nitrocellulose membrane (Bridge of Weir, United Kingdom), which were incubated with suitable primary and secondary antibodies for the determination of each target protein and parameter of interest, to know: HSP25 (Enzo Life Sciences, #ADI-SPA-801), HSP60 (Enzo Life Sciences, #ADI-SPA-806), HSP70 (Enzo Life Sciences, #ADI-SPA-810), HSP90 (Enzo Life Sciences, #ADI-SPA-831), SOD (Abcam, #AB51254), and Catalase (Abcam, #AB1877). Bands obtained were observed by chemiluminescence [UVITEC Cambridge (Model Alliance LD2)], and band intensity (semi-quantitative or quantitative evaluation) was determined with the ImageJ software (v. 1.44 for Windows).

### Statistical Analyses

The Kolmogorov–Smirnov test was used to check if data obtained presented a normal distribution. Body weight, hematological and biochemical parameters were analyzed by ANOVA, followed by Tukey’s test. For checking significant differences regarding proteins of defense and antioxidant systems (Western Blot technique), the Ducan *post hoc* test was applied. For all tests performed, the significance level was set at 5% (*p* < 0.05). GraphPad Prism 7.0 software was used to carry out all statistical analyses (GraphPad Software Inc., San Diego, United States).

## Results and Discussion

This study aimed to evaluate the production of a probiotic “requeijão cremoso” incorporated with *B. coagulans* GBI-30 6086 and to assess the metabolic effect of its consumption in an animal model. The main findings of this research show that the step of incorporation of *B. coagulans* GBI-30 6086 into the “requeijão cremoso” processed cheese did not affect the maintenance of the metabolic homeostasis of rats. The final product showed stable probiotic counts and better homogenization regardless of the probiotic incorporation step.

As seen in Table 2, “requeijão cremoso” with and without probiotic presented similar nutritional composition. Cheeses are well-known as efficient matrices for carrying probiotic bacteria because high levels of lipids and the proteins protect strains throughout the gut (Cruz et al., 2009; Lollo et al., 2015; Martins et al., 2018).

**TABLE 2:** Average values (±standard deviation) of different groups of substances evaluated for centesimal composition determined in commercial and experimental diets offered to animals monitored in the study.

**(g/100 g)**	**Control “requeijão cremoso”^∗^**	**Probiotic “requeijão cremoso”^#^**
Humidity	56.7 ± 0.9	55.7 ± 0.5
Lipids	19.5 ± 1.2	20.7 ± 1.0
Protein	19.1 ± 0.4	18.6 ± 0.4
Ash content	2.5 ± 1.1	2.7 ± 0.6
Carbohydrates	2.2 ± 0.7	2.3 ± 0.9
Total	100	100

*^∗^Control –“requeijão cremoso” without the addition of *B. coagulans* GBI-30 6086); ^#^Probiotic “requeijão cremoso” – “requeijão cremoso” containing *B. coagulans* GBI-30 6086 inoculated after fusion stage.*

**FIGURE 2:**
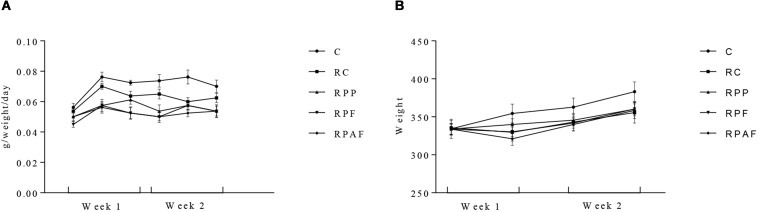
Body weight gain **(A)** and commercial diet intake **(B)** in 40 male Wistar rats, randomly assigned to five experimental groups over 2 weeks. Control (C); Control “requeijão cremoso” cheese (RC); Probiotic “requeijão cremoso” containing spores of *B. coagulans* GBI-30 6086 inoculated in milk pasteurization step (RPP); Probiotic “requeijão cremoso” containing spores of *B. coagulans* GBI-30 6086 inoculated during fusion stage (RPF); Probiotic “requeijão cremoso” containing spores of *B. coagulans* GBI-30 6086 inoculated after fusion stage (RPAF).

Initial counts of *B. coagulans* GBI-30 6086 observed in the “requeijão cremoso” on the first day of the experiment were 7.8 × 10^5^; 5.5 × 10^7^ and 1.4 × 10^7^ spores/g for RPP, RPF and RPAF, respectively. After 2 weeks, the counts of *B. coagulans* GBI-30 6086 determined in the food product were, respectively, 2.0 × 10^4^, 2.8 × 10^7^ and 1.2 × 10^7^ spores/g for RPP, RPF and RPAF. The high resistance of spores is demonstrated by the fact that the decrease in their counts was less or equal to 1 log. This result shows that the microorganism remained viable (stable spores’ populations) during the entire experimental period. All animals presented body weight gain throughout the 2 week period of tests (Figure 2). Animals fed with “requeijão cremoso” (RC, RP, RF, and RPF) consumed less amounts of commercial feed compared to control group (C). This fact may have occurred due to a greater satiety of the animals that received the “requeijão cremoso” since it has high viscosity and could have led to an extended period for gastric emptying (Mourão and Bressan, 2009). Also, the “requeijão cremoso” is rich in proteins and lipids, approximately 40% in total, which may also have contributed to the satiety of the animals (Pedrosa et al., 2009). Therefore, differences observed in the amount of food intake cannot be attributed to nutritional deficiency.

**FIGURE 3:**
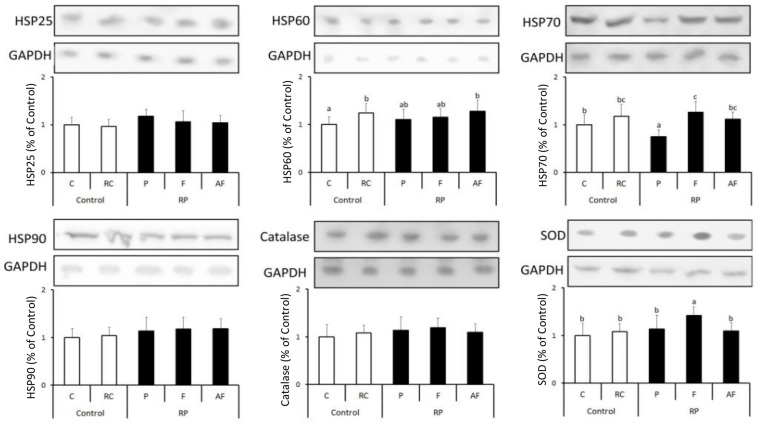
Means and standard deviations of the expression of HSP25, HSP60, HSP70, HSP90, Catalase and SOD determined using the Western blot technique. Control (C); Control “requeijão cremoso” (RC); Probiotic “requeijão cremoso” containing spores of *B. coagulans* GBI-30 6086 inoculated in milk pasteurization step (RPP); Probiotic “requeijão cremoso” containing spores of *B. coagulans* GBI-30 6086 inoculated during fusion stage (RPF); Probiotic “requeijão cremoso” containing spores of *B. coagulans* GBI-30 6086 inoculated after fusion stage (RPAF). Different letters represent a significant difference (*p* < 0.05) between the groups.

Figure 3 shows the results obtained for the quantification of different anti-stress and antioxidant proteins in the five groups of animals studied. Levels of stress proteins determined were kept stable in all animal groups tested, indicating a balance in the homeostasis of the cellular metabolism (Figure 3).

Overall, levels of HSP70 were shown to be similar between groups C and those treated with the probiotic strain. The only exception was related to animals belonging to the group that received the “requeijão cremoso” with *B. coagulans* GBI-30 6086 added before pasteurization (RPF). In this group, a significant increase (*p* < 0.05) on HSP70 expression was observed in comparison to C group. Bearing in mind that animals were healthy, a possible justification for these results would be the lower counts of viable spores found in the probiotic “requeijão cremoso” resulting in a lower expression of anti-stress proteins in the RPP group animals. HSP70 is particularly associated with the aid of the antioxidant system, protecting other proteins from possible damages and denaturation (Silver and Noble, 2012; de Moura et al., 2013). According to our results, HSP70 result did not have a concomitant effect on the expression of catalase enzyme. Nonetheless, higher levels of both SOD and HSP70 expression were observed in animals fed with “requeijão cremoso” containing *B. coagulans* GBI-30 6086 added in the fusion stage (RPF) in comparison to other groups of animals studied. These findings suggest that heat treatment and micro environmental conditions used in the fusion stage may have worked as determinant factors for a greater germination of the spores and, therefore, a higher expression of the enzyme that plays a key role against free radical compounds (Aguiló et al., 2005).

These observations are consistent with the results obtained by the analysis of immune system cells (Tables 3, 4), which counts remained within normal ranges, indicating health condition in animals studied. Finally, a significant increase (*p* < 0.05) in HSP60 expression was observed by consumption of the “requeijão cremoso” in comparison to C group, although no major differences had been observed with the incorporation of *B. coagulans* GBI-30 6086.

**TABLE 3:** Mean values (±SD) of Eritogram from hematological parameters.

**Hematological parameters**				
**Feeding type**	**Eritogram**			
	
	**Red blood cell (10^6^ cel/μL)**	**Hemoglobin (g/dL)**	**Hematocrit (%)**	**Platelets (10^6^ cel/μL)**
Control	7.6 ± 2	14.14 ± 0.96	43.5 ± 1	1 ± 1
RC	8.50 ± 2.49	15.85 ± 1.32	43.71 ± 1.95	1.25 ± 1.40
RPP	8.19 ± 4.02	17.29 ± 0.87	41.43 ± 2.03	1.22 ± 1.72
RPF	8.60 ± 2.64	15.25 ± 0.92	41.88 ± 1.22	1.08 ± 3.46
RPAF	8.39 ± 4.78	13.25 ± 0.96	41.63 ± 2.10	1.06 ± 3.01

*Diets: Control (C); Control (RC); Probiotic “requeijão cremoso” containing *B. coagulans* GBI-30 6086 inoculated in milk pasteurization step (RPP); Probiotic “requeijão cremoso” containing *B. coagulans* GBI-30 6086 inoculated during fusion stage (RPF); Probiotic “requeijão cremoso” containing *B. coagulans* GBI-30 6086 inoculated after fusion stage (RPAF). Statistical test (One way ANOVA).*

**TABLE 4:** Mean values (±SD) of Leukogram from hematological parameters.

**Hematological Parameters**						
**Feeding type**	**Leukogram**					
	
	**Leukocytes (10^3^ cel/μL)**	**Lymphocyte (10^3^ cel/μL)**	**Eosinophil (cel/μL)**	**Monocyte (cel/μL)**	**Neutrophil (10^3^ cel/μL)**	**Basophile (cel/μL)**
Control	12.60 ± 6589	7.42. ± 3438	262 ± 317	1008 ± 653	3.91 ± 3284	0
RC	13.06 ± 5122	8.75. ± 3353	363 ± 330	590 ± 715	3.36 ± 3239	0
RPP	12.53 ± 3351	7.30. ± 3006	299 ± 249	421 ± 485	4.52 ± 3092	0
RPF	13.33 ± 911	8.76 ± 2702	350 ± 204	804 ± 456	3.41 ± 2896	0
RPAF	13.99 ± 4353	9.70 ± 2481	417 ± 208	574 ± 444	3.31 ± 782	0

*Diets: Control (C); Control (RC); Probiotic “requeijão cremoso” containing *B. coagulans* GBI-30 6086 inoculated in milk pasteurization step (RPP); Probiotic “requeijão cremoso” containing *B. coagulans* GBI-30 6086 inoculated during fusion stage (RPF); Probiotic “requeijão cremoso” containing *B. coagulans* GBI-30 6086 inoculated after fusion stage (RPAF). Statistical test (One way ANOVA).*

The quantification of HSPs has a predictive value of animal’s health status since it indicates the homeostatic state of the organism. In fact, for animal health-model studies, the evaluation of HSPs levels can be used to correlate optimal values and beneficial probiotic effects, and also for the determination of intracellular homeostasis parameters.

The ingestion of probiotics, particularly those belonging to genera *Bifidobacterium* and *Lactobacillus* can modulate the levels of oxidation of the organism (Jensen et al., 2010). This modulation occurs due to the fact these microorganisms can induce antioxidant reactions, inhibiting the formation of reactive oxygen metabolites in the gut (Amaretti et al., 2013). Hereof, according to an *in vitro* study published by Jensen et al. (2010), *B. coagulans* GBI-30 6086 was able to modulate the release of anti-inflammatory by intestinal epithelial cells, highlighting the potential of this strain to exert beneficial effects on human health (Jensen et al., 2010).

Considering that HSPs are stress-indicating proteins, the protective role of probiotics can be mediated, in part, by facilitation of HSP expression under stressful conditions. For instance, if a host is exposed to a certain situation that can disrupt its homeostasis but its GIT is previously colonized by probiotics these beneficial microorganisms can stimulate the production of HSPs, which interact with the immune system leading to the production of defense cells and controlling the inflammatory process (Coppola and Gil-Turnes, 2004; Taverniti and Guglielmetti, 2011; Liu et al., 2014).

It is known that the expression of HSPs is a partial stress-conditioned phenomena (Santos-Junior et al., 2018). Thus, in the present study, since animals tested were only transiently colonized by *Bacillus* spp. due to a short period of its consumption and also not stressed, this phenomenon could had been attenuated resulting in minor differences observed between treatments (Elshaghabee et al., 2017).

**FIGURE 4:**
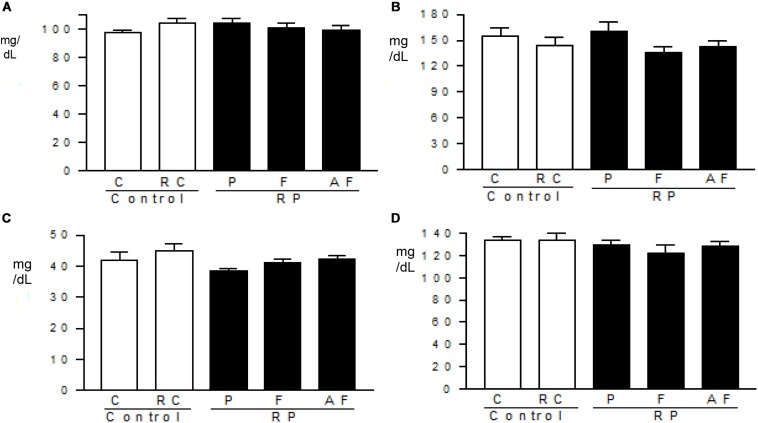
Metabolic profile. Mean values and standard deviations of total cholesterol **(A)**, triglycerides **(B)**, HDL-cholesterol **(C)** and glucose **(D)** determined in blood samples. Control (C); Control “requeijão cremoso” (RC); Probiotic “requeijão cremoso” containing spores of *B. coagulans* GBI-30 6086 inoculated in milk pasteurization step (RPP); Probiotic “requeijão cremoso” containing spores of *B. coagulans* GBI-30 6086 inoculated during fusion stage (RPF); Probiotic “requeijão cremoso” containing spores of *B. coagulans* GBI-30 6086 inoculated after fusion stage (RPAF).

Figure 4 shows no significant alteration (*p* > 0.05) in lipid profile and glucose levels amongst the groups tested. These findings indicate that the “requeijão cremoso” added of *B. coagulans* GBI-30 6086 did not induce alterations in the energy metabolism.

The average values determined for total cholesterol (98.9–110.2 mg/dL), HDL-cholesterol (11.4–20.4 mg/dL), triglycerides (110–174.8 mg/dL) and glycemia (150.7–207.5 mg/dL) in blood samples were within the limits of the reference ranges recommended by the Centro de Bioterismo – Faculty of Medicine of the University of São Paulo (FMUSP, 2008). The analysis of the lipid profile is important for the evaluation of the risk of developing chronic illnesses such as dyslipidemia and cardiovascular diseases. Total cholesterol, triglycerides, and HDL are biochemical markers easily altered by diet. Deregulation of these markers can lead to metabolic disorders in the long run (Castro et al., 2004; Zambon et al., 2009).

**FIGURE 5:**
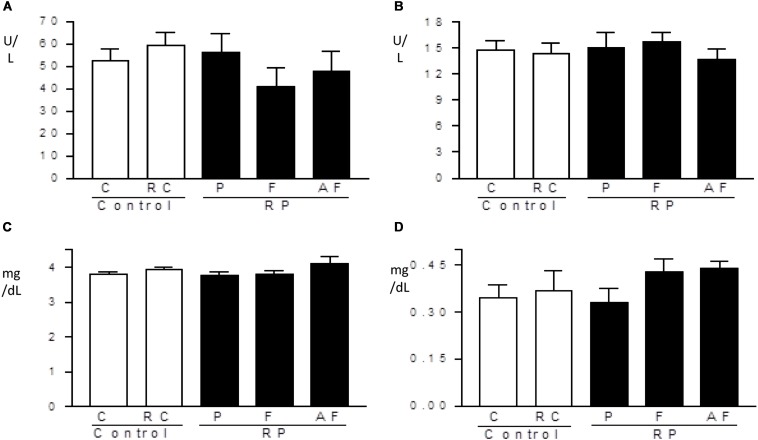
Hepatic and renal profiles. Mean values and standard deviations of AST: aspartate aminotransferase **(A)**, ALT: alanine aminotransferase **(B)**, uric acid **(C)** and creatinine-kinase **(D)** determined in blood samples. Control (C); Control “requeijão cremoso” (RC); Probiotic “requeijão cremoso” containing spores of *B. coagulans* GBI-30 6086 inoculated in milk pasteurization step (RPP); Probiotic “requeijão cremoso” containing spores of *B. coagulans* GBI-30 6086 inoculated during fusion stage (RPF); Probiotic “requeijão cremoso” containing spores of *B. coagulans* GBI-30 6086 inoculated after fusion stage (RPAF).

Enzyme levels of the hepatic system and biochemical markers of the renal system were not altered by ingestion of the “requeijão cremoso” containing *B. coagulans* GBI-30 6086 (Figure 5). All treated groups showed similar values in comparison to C group. Levels obtained for hepatic enzymes were lower than the reference range used by FMUSP (2008) (114.0–290.8 U/L for ALT and 129.0–148.1 U/L for AST). Such differences may be related to the genetic variability of the animals, environmental influence or differences in the manipulation of the rats, as pointed out by other investigators (Santos et al., 2010; Lima et al., 2014). Levels of creatine and uric acid determined in the present study were similar to the reference values used by Lima et al. (2014), to know: 0.24–1.20 mg/dL for creatine and 1.00–3.20 mg/dL for uric acid, and no differences were observed between groups.

**FIGURE 6:**
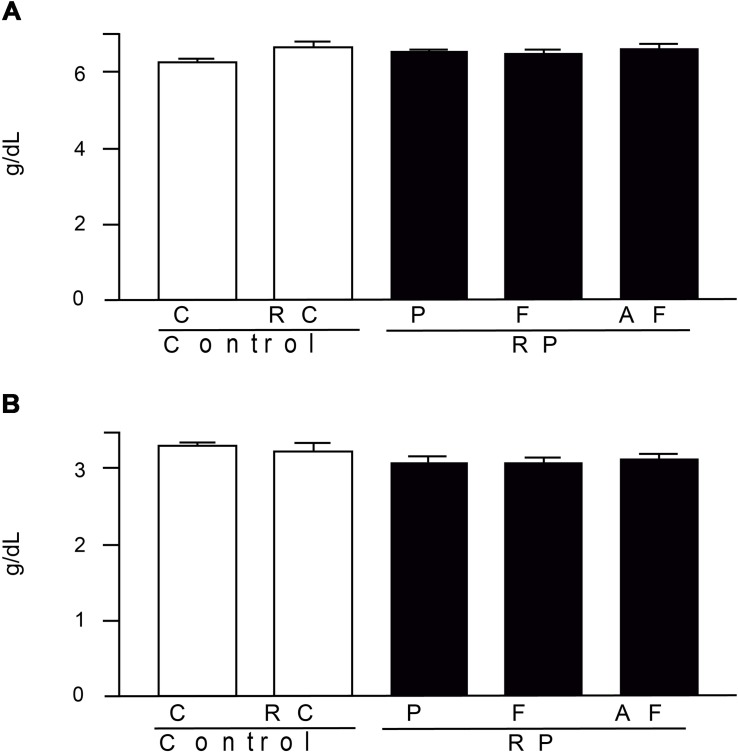
Mean values and standard deviations of total proteins **(A)** and blood albumin **(B)** determined in blood samples. Control (C); Control “requeijão cremoso” (RC); Probiotic “requeijão cremoso” containing spores of *B. coagulans* GBI-30 6086 inoculated in milk pasteurization step (RPP); Probiotic “requeijão cremoso” containing spores of *B. coagulans* GBI-30 6086 inoculated during fusion stage (RPF); Probiotic “requeijão cremoso” containing spores of *B. coagulans* GBI-30 6086 inoculated after fusion stage (RPAF).

The results obtained for total protein and albumin (Figure 6), are within the respective ranges of reference values adopted by FMUSP laboratory (FMUSP, 2008) (5.5–10.4 g/dL and 2.8–6.1 g/DL, respectively). Hence, the studied groups did not differ (*p* > 0.05), demonstrating that the nutritional status of the animals was not altered due to the diet. Abnormal total protein values, in turn, indicate nutritional problems, renal or hepatic disease.

Resemblance between reference values for animals and humans is due to their physiological similarity. For example, the ideal level for total proteins in humans is in the range of 6–8.3 g/dL, close to those observed in the rodents evaluated in our study (6.29–6.65 g/dL). The uric acid is naturally produced by the body through the breakdown of proteins (purines) mainly obtained from the diet and further eliminated by the kidneys. In humans, the ideal level of uric acid is in the range of 3.4–7 g/dL, a value similar to the ones found in the present study (3.74–4.29 g/dL).

The results of the hematological, metabolic and immune analyses were within health reference values and showed no difference between treatments (*p* > 0.05). Different results were observed by Lollo et al. (2013a, b). According to these authors, rats submitted to strenuous physical exercise presented impaired functions of the immune system. These functions, however, were re-established after ingestion of probiotic yogurt containing *Bifidobacterium longum* BL 05 and *Lactobacillus acidophilus* LA 14. Reference values for hematological parameters vary significantly according to race, lineage, age, sex and health status of the animal, as well as they may undergo changes related to blood manipulation and analysis. The probiotic “requeijão cremoso” should be tested under a highly stressful environment, to verify the similarity of probiotic effect reported by these authors.

Neutrophil is considered a quick and easy marker to quantify stress intensity and indicate inflammatory status (Zahorec, 2001). Increased numbers of neutrophils and lymphocytes in the bloodstream are indicative of inflammatory and infectious states (Lollo et al., 2013b). According to the evaluation of different markers of the defense system, the values observed in the leukogram of the animals studied highlight no major modifications in the levels of stress proteins, indicating that the dietary food matrix tested (“requeijão cremoso”) added of the probiotic strain did not impair either homeostasis or health status of the rats. Therefore, our hematological and biochemical data suggest *B. coagulans* GBI-30 6086 is safe and did not cause any harm, inflammation or signs suggestive of allergenicity in animals tested during the study.

## Conclusion

The incorporation of *B. coagulans* GBI-30 6086 in the “requeijão cremoso” was successfully achieved and the probiotic strain remained viable in all steps tested. In reality, the incorporation of the spores in the fusion stage facilitated the technological process enabling a better homogenization of the product and could possibly avoid recontamination of the product after heat treatment. Probiotic counts determined in the final product were similar to those observed at different fusion steps throughout the 2-week period of animal experiments. This demonstrates the high resistance of *B. coagulans* GBI-30 6086 against high temperatures and indicates its great potential to be incorporated in food technological processes. Moreover, the incorporation of the probiotic strain in different steps of the “requeijão cremoso” did not affect the maintenance of the metabolic homeostasis of rats. Thus, new researches could explore the development of new products added of *B. coagulans* GBI-30 6086 and the evaluation of distinct HSPs and anti-oxidant enzymes. Further efforts can be made to determine the effects of the consumption of *B. coagulans* GBI-30 6086 in the long term as well as to enhance its functionality by testing other food matrices.

## Data Availability Statement

The datasets generated for this study are available on request to the corresponding author.

## Ethics Statement

This study was carried out in accordance with the recommendations of Ethical Principles for Animal Experimentation – Brazilian Association of Laboratory Animal Science (SBCAL) and the federal regulation Law n. 11.794, October 08, 2008, and Decree n. 6.899, July 15, 2009. The protocol was approved by the Animal Research Ethics Committee (CEUA/UNICAMP, protocol no. 3444-1).

## Author Contributions

MS, AC, PL, and AS were responsible for the study design. MS reviewed the literature, performed both the experiments and data analysis, and prepared the draft of the manuscript. VS-J contributed on the analysis of biochemical, hematological and immunoblotting data, and writing of the manuscript. ET helped on data treatment and writing of the manuscript. PL, JA-F, and PM assisted on the development of the animal experimental protocol and methods used, and helped on data interpretation. JA-F revised the draft manuscript and made suggestions to enhance its readability and scientific content. EP and CB helped on the performance of the experiments, including formulation and preparation of “requeijão cremoso” containing the probiotic microorganism, including all pre-experimental tests needed to ensure a substantial study, and contributed on writing and review of the manuscript. RM co-supervised the work and assisted on preparation and review of the manuscript. AS supervised the work, obtained funds its completion, and contributed on writing and review of the manuscript. All authors critically reviewed the manuscript and approved the final version.

## Conflict of Interest

The authors declare that the research was conducted in the absence of any commercial or financial relationships that could be construed as a potential conflict of interest.
